# Alpha-2 adrenergic stimulation triggers Achilles tenocyte hypercellularity: Comparison between two model systems

**DOI:** 10.1111/j.1600-0838.2011.01442.x

**Published:** 2012-01-31

**Authors:** L J Backman, G Andersson, G Fong, H Alfredson, A Scott, P Danielson

**Affiliations:** 1 Department of Integrative Medical Biology, Anatomy, Umeå UniversityUmeå, Sweden; 2 Department of Surgical and Perioperative Sciences, Sports Medicine, Umeå UniversityUmeå, Sweden; 3 Department of Physical Therapy, University of British ColumbiaVancouver, British Columbia, Canada; 4 Centre for Hip Health and Mobility, Vancouver Coastal Health and Research InstituteVancouver, British Columbia, Canada

**Keywords:** Tendinopathy, α_2A_ adrenoreceptor, Tendinosis, Achilles tendon

## Abstract

The histopathology of tendons with painful tendinopathy is often tendinosis, a fibrosis-like condition of unclear pathogenesis characterized by tissue changes including hypercellularity. The primary tendon cells (tenocytes) have been shown to express adrenoreceptors (mainly alpha-2A) as well as markers of catecholamine production, particularly in tendinosis. It is known that adrenergic stimulation can induce proliferation in other cells. The present study investigated the effects of an exogenously administered alpha-2 adrenergic agonist in an established *in vivo* Achilles tendinosis model (rabbit) and also in an *in vitro* human tendon cell culture model. The catecholamine producing enzyme tyrosine hydroxylase and the alpha-2A-adrenoreceptor (α_2A_ AR) were expressed by tenocytes, and alpha-2 adrenergic stimulation had a proliferative effect on these cells, in both models. The proliferation was inhibited by administration of an α_2A_ AR antagonist, and the *in vitro* model further showed that the proliferative alpha-2A effect was mediated via a mitogenic cell signaling pathway involving phosphorylation of extracellular-signal-regulated kinases 1 and 2. The results indicate that catecholamines produced by tenocytes in tendinosis might contribute to the proliferative nature of the pathology through stimulation of the α_2A_ AR, pointing to a novel target for future therapies. The study furthermore shows that animal models are not necessarily required for all aspects of this research.

Tendinopathy is a chronic condition of tendon pain and thickening characterized by structural and fibrotic changes of the tendon (tendinosis) and the surrounding tissues. The microscopic tissue changes seen in tendinosis include hypercellularity, vascular proliferation, and structural deterioration of the tendon itself (Khan et al., 1999). Paratendinous tissues display increased blood flow in Achilles tendinosis patients (Astrom & Westlin, 1994; Ohberg et al., 2001). It has been suggested that this increased blood flow and vascular proliferation is accompanied by sprouting of sensory nerve fibers within the tendon (Schubert et al., 2005), although this has not been confirmed by other studies (Bjur et al., 2005; Andersson et al., 2007). In fact, the paratendinous region ventral to the Achilles tendon, i.e., an area often displaying the most prominent increase in blood flow, is primarily innervated by sympathetic nerve fibers (Andersson et al., 2007).

The mechanisms of persistent tendon pain in tendinosis are not fully understood. The role of sympathetic nerves in the ventral paratendinous tissue of the Achilles tendon is also unclear, although this area is of particular interest as it has been targeted in some treatment regimens (Alfredson et al., 2007; Alfredson, 2011). In studies on other tissues and pain disorders, it has been shown that the peripheral sympathetic nervous system plays a role in neurogenic inflammation (Kim et al., 1998, 1999; Souza et al., 2005). The sympathetic nervous system can also generate or enhance pain (e.g., Baron et al., 1999). Furthermore, norepinephrine can stimulate proliferation of myofibroblastic hepatic stellate cells in mice and induce collagen gene expression in such cells (Oben et al., 2003). Interestingly, in the clinical treatment situation, when interfering with the sympathetic nerve rich paratendinous region, by sclerosing injections (Alfredson et al., 2007) or surgical scraping (Alfredson, 2011), there is a short-term vascular response (days) inside the tendon with a clearly visible increased blood flow and eventually a longer-term normalization of the tendon structure and thickness.

In view of the above, the possible influence of the adrenergic system in the development of tendon pain and tissue pathology has been the focus of recent studies. Several structures in human tendons are indeed susceptible to adrenergic stimulation in the sense that they express adrenergic receptors. Adrenoreceptors have been detected on blood vessels (mainly arterioles) and nerve structures within tendons – in the latter case the receptors sometimes being found in the same fascicles as sensory neurons (Danielson et al., 2007b; Bjur et al., 2008), which is of interest since pain induced or sustained by sympathetic neurotransmitters has been proposed to be mediated via adrenergic receptors on sensory afferents (Baron, 2000). In addition, the primary tendon cells, the tenocytes, express adrenoreceptors *in vivo*, in particular the alpha-2A-adrenoreceptor (α_2A_ AR) (Danielson et al., 2007b), making them possible targets for sympathetic neurotransmitters. Hypothetically, such transmitters might not only derive from the peripheral sympathetic nerve system or the blood circulation, but also from the tendon cells themselves; this is suggested by the localization of tyrosine hydroxylase (TH; the rate-limiting enzyme in catecholamine synthesis) in tenocytes. Most interestingly, this enzyme (TH) is more highly expressed in tendinosis (Danielson et al., 2007a, b2007b; Bjur et al., 2008). α_2A_ AR is a G-protein coupled receptor (GPCR), and GPCRs are known to be involved in transducing extracellular signals leading to proliferation via activation of mitogen-activated protein kinases (MAPKs) (Koon et al., 2004; Yamaguchi et al., 2005) like the extracellular-signal-regulated kinases 1 and 2 (ERK1/2), which could influence the rate of tenocyte proliferation.

We have recently validated an *in vivo* model (rabbit) for Achilles tendon overuse (Andersson et al., 2011b), based on earlier studies (Backman et al., 1990, 1991). This model produces tendinosis-like tissue changes, such as tenocyte proliferation and angiogenesis, in the Achilles tendon after 3 weeks of overuse (protocol: 150 dorsi-/plantarflexions of the ankle joint per minute for 2 h every second day), but not after a single week of overuse (Andersson et al., 2011b). Furthermore, the changes are also seen on the contralateral (unstimulated) side. This model provides an opportunity to intervene during the development of tendinosis, and test hypotheses regarding pathogenesis (Andersson et al., 2011a). However, due to ethical considerations, alternative models are desired, and it would also be beneficial to study the behavior of human tendon cells, as compared with those of animals, and we have therefore established an *in vitro* model of primary cell cultures of human tendon derived cells in our laboratory (Backman et al., 2011) as an alternative. These two model systems (*in vitro* and *in vivo*) have, however, not yet been compared with regard to the adrenergic system.

The overall goal of the present study was to better understand the adrenergic system in tendon tissue and its potential role in the development of tendinosis. The specific aims of the study were to investigate (a) whether the rabbit Achilles tendon tissue expresses TH and the α_2A_ AR *in vivo* and also if this is present in human tendon derived cells *in vitro*; (b) whether an exogenously administered α_2_ agonist (clonidine) or antagonist (BRL 44408 Maleate) influences proliferation of tenocytes *in vivo*, in the rabbit model, and/or of human tendon derived cells *in vitro*; and finally (c) whether an *in vitro* model of culturing human tendon cells is appropriate to reduce the use of an established animal model for this line of inquiry. The latter aim is part of the drive to reduce animal research where it is not absolutely necessary.

## Methods

### *In vivo* model

#### Animals

Twenty-four adult female New Zealand white rabbits aged 6–9 months were used. The age difference was due to the varying time required to reach a weight of approximately 4 kg. Rabbits were randomly divided into four equally sized treatment groups (*n* = 6). The first group (“uninjected controls”) was used as a control group which did not receive any additional treatment aside from the ordinary training schedule to the right limb of the animal (described below), the second group (“clonidine injected”) received paratendinous injections of the α_2_ agonist clonidine, the third group (“BRL injected”) was given paratendinous injections of a specific antagonist for the α_2A_ AR (BRL 44408 Maleate), and the fourth group received paratendinous injections of isotonic saline (“NaCl controls”). The experiments ran simultaneously with another reported study of ours, investigating the effect of the neuropeptide substance P (Andersson et al., 2011a), and the same control groups (“uninjected controls” and “NaCl controls”) were used in both studies for the ethical reason that it reduced the number of rabbits needed for the studies. In the “clonidine injected” group and the “BRL injected” group, injections were given to both the exercised (right) and unexercised (left) limb for comparison.

#### Experimental design

The animal overuse model used in this paper has been thoroughly described earlier (Backman et al., 1990; Andersson et al., 2011a, b2011b). Using a pneumatic piston, it produces a passive dorsiflexion and plantar flexion of the right ankle of the hind leg (150 movements/min), which is synchronized with a concentric muscle contraction of the triceps surae muscle of the same leg via electrical stimulation over the gastrocnemius motor point in the plantar flexion phase. The contralateral, left leg was kept at rest. In order to restrict excessive movement in the hip and knee of the resting (left) leg, a band was fastened around the pelvis of the rabbits. The experiment ran for 2 h every second day for a total of 1 week (four sessions). In between sessions they were housed at a 12 h day/night cycle in spacious cages allowing freedom of movement with food and water *ad libitum*. Injections of 0.2–0.3 mL/kg of fentanylfluanison (Hypnorm) in combination with diazepam 5 mg/mL (0.2 mL/kg) was given as anesthesia. The fentanylfluanison was boosted by an additional injection of 0.1 mL/kg every 30–45 min to maintain sedation. Post exercise, buprenorophine (Temgesic; 0.01–0.05 mg/kg) was administered subcutaneously. Experiments were approved by the local ethical committee for research on animals.

#### Injection treatments

In combination with the training regime, rabbits were given paratendinous injections after each session. 400 μg/mL clonidine (c7897; Sigma, Saint Louis, MO, USA) an α_2_ adrenergic agonist commonly added to local anesthetics, was given in 1 mL of isotonic saline to the “clonidine injected” group. The same amount (400 μg/mL in 1 mL of isotonic NaCl) was given of the specific antagonist for the α_2A_ AR (BRL 44408 Maleate, Tocris Bioscience, Bristol, UK) to the “BRL injected” group. The third injection group was given 1 mL of isotonic saline (0.9% NaCl). The injections were all directed to the area ventral to the Achilles tendon proper; this tissue has been shown to display marked vascularity (Ohberg et al., 2001) as well as a predominantly sympathetic innervation (Andersson et al., 2007) in patients with Achilles tendinosis. The “clonidine injected” and “BRL injected” groups were given bilateral injections. Due to anesthesia side effects, one rabbit was lost in the “NaCl control” group.

#### Sampling, fixation and sectioning

The rabbits were anesthetized with sodium pentobarbitol (60 mg/kg i.p.) and sacrificed by a subsequent overdose one day after the last training session. Achilles tendons and the triceps surae muscle of both hind legs were transported from the animal facility to the lab where biopsies were collected. The Achilles tendon was dissected into three separate parts: one part close to the myotendinous junction, one from the mid-part, and one from within close proximity of the calcaneal insertion. The biopsies were approximately 5 × 5 mm each. Following dissection, samples were mounted transversely with optimal cutting temperature compound (TissueTek, Miles Laboratories, Naperville, IL, USA). These were then frozen in isopropane-chilled liquid nitrogen and stored at −80 °C. None of the samples were chemically fixed. A cryostat was used to cut 7 μm sections for immunohistochemistry and 10 μm for *in situ* hybridization.

### *In vitro* model

#### Isolation of tendon cells from human subjects

Samples of human Achilles tendon tissue were obtained under sterile conditions from healthy donors who had given informed consent. Donors had no history of Achilles tendon pain and the tendons were assessed as normal using color Doppler ultrasound examination. The study was approved by the Regional Ethical Review Board in Umeå and was performed according to the principles of the Declaration of Helsinki. For further details see Backman et al. 2011.

#### Cell culturing

Primary human tendon derived cell cultures were established as previously described (Backman et al., 2011). In brief, the samples were carefully washed and minced, then enzymatically digested at 37 °C using collagenase (Clostridopeptidase A, C-0130 Sigma) diluted in Dulbecco's Modified Eagle Medium (D-MEM). Cells were grown in D-MEM supplemented with 10% fetal bovine serum (FBS), 1% pen-strep, and 0.2% L-Glutamine, all from Invitrogen (Invitrogen, Paisley, UK). All experiments were performed in serum-starved conditions of 1% FBS.

### Immunocyto-/histochemistry

Immunocyto-/histochemistry were performed in order to detect TH and α_2*A*_ AR, as well as to facilitate the evaluation of vascular structures (CD31-staining) in the tissue sections. The sections to be stained for TH and α_2*A*_ AR were pretreated with acid potassium permanganate for 2 min to enhance immunofluorescence and thereafter rinsed three times in phosphate buffered saline (PBS). The sections were then incubated in 1% Triton X-100 (Merck, Darmstadt, Germany) for 20 min, and then incubated in 5 % normal swine serum and 0.1% bovine serum albumin for 15 min. The primary antibody was administered, and incubation was done overnight in a humid chamber kept at 4 °C. On the second day, washing was done three times in PBS before the sections were incubated in 5% normal swine serum once again and then incubated with a secondary antibody in the form of tetramethylrhodamine isothiocyanate (TRITC) conjugated swine anti-rabbit IgG (code: R0156; Dako, Glostrup, Denmark) or fluorescein isothiocyanate conjugated swine anti-rabbit IgG (code: F0205; Dako), 1:40 for 30 min at 37 °C. Staining for CD 31 was performed in the same way, except normal rabbit serum was utilized instead of swine serum, and the secondary antibody used was a TRITC-conjugated rabbit anti-mouse IgG (code: R0270; Dako), used at the same concentrations as above.

The primary antibody used to detect TH was supplied by Pel-Freez (P40101, Pel-Freez, Rogers, AR, USA) and used at a concentration of 1:100, the α_2*A*_ AR antibody was supplied from Oncogene (PC161 Oncogene, Boston, MA, USA), diluted to 1:50. Both these antibodies were of rabbit polyclonal origin. A mouse monoclonal antibody directed toward CD31, was used to visualize the vascular structures (M0823, Dako, Glostrup, Denmark).

For the immunocytochemistry of the cell cultures, a fixation in 2% paraformaldehyde was done before the immunostaining, i.e., cells were not pretreated with acid potassium permangante or permeabelized in 1% Triton X-100. In the final step the mounting was done with 4′,6-diamidino-2-phenylindole (DAPI) to stain the nuclei of the cells. The protocol for these cells was otherwise the same as for the rabbit tissue sections as described above.

### *In situ* hybridization

A custom designed antisense digoxigenin (DIG)-hyperlabeled oligonucleotide probe was used to detect mRNA for TH (GD1811-OP, GeneDetect, Auckland, New Zealand). The probe was used at 50 ng in 15 μL of hybridization solution. The antisense probe sequence was: AACCGCGGGGACATGATGGCCT. For control purposes, a negative control using the corresponding sense DIG-hyperlabeled ssDNA probe was used, and positive controls in the form of a Poly(dT) probe (GD4000-OP; GeneDetect) and a β-actin antisense probe (GD5000-OP, GeneDetect) were used. For detection, an alkaline phosphatase labeled anti-DIG antibody (11 093 274 910; Roche, Mannheim, Germany) was used.

For further information on the *in situ* hybridization method, please see Danielson et al., 2007a, as well as the original protocol upon which ours is based (Panoskaltsis-Mortari & Bucy, 1995).

### Histological evaluation of the rabbit tendon tissue (including evaluation of cell and vascular proliferation)

Sections of the three parts of the tendon (cf. “Sampling, fixation and sectioning”) were stained using hematoxylin-eosin and evaluated for general morphology, primarily examining the pattern of collagen organization and tenocyte morphology, and detailed tendon cell quantification was made by one of the researchers (G. A.) according to an established method used previously (Andersson et al., 2011a, b2011b). In short, this was done by counting the number of cells in three randomly selected micrographs (283 × 213 μm^2^) for each of the three parts of the tendon, from which a mean was calculated. After controlling that there was no statistically significant difference between the three tendon parts, a mean value for the whole tendon was used in further calculations. The data for the uninjected controls and the NaCl controls were derived from the previous study (Andersson et al., 2011a, b2011b), cf. “Animals.”

The grading for vascularity was done in the same manner as in the previous studies (Andersson et al., 2011a, b2011b) and is based on a modified variant of the Bonar scale for tendinosis evaluation (Cook et al., 2004). Each part of the tendon was given a grade of 0–3 and then combined to give each tendon a final grade of 0–9; 0 being normal and 9 being highly vascularized. One researcher (G. A) graded the vascularity in a blinded fashion.

The test–retest reliability for this researcher has been quantified previously as being good (Andersson et al., 2011b).

### Western blot analysis

Cells were lysed in buffer [150 mM Sodium chloride, 1% Triton, 0.5% Sodium deoxycholate, 0.1% Sodium Dodecyl Sulphate (SDS), 50 mM Tris, pH 8.0]. Protein determination was done and equal amount of cell extracts were separated on a SDS-polyacrylamide gel electrophoresis and blotted to polyvinylidene fluoride membranes. Membranes were blocked in 5% dried milk in Tris-buffered Saline Tween-20 and proteins were detected with a rabbit monoclonal anti-human phospho-p44/42 MAP kinase (Cell signal, Danvers, MA, USA; code: 4370) at a concentration of 1:2000, a rabbit polyclonal Anti-TH (code: p40101; Pel-Freez, Rogers, AR, USA) at a concentration of 1:2000, and a rabbit polyclonal beta-actin (Cell signal; Code: 4967) at a concentration of 1:2000. Membrane was incubated with secondary goat anti-rabbit IgG antibody conjugated to horseradish peroxidase and visualized by employing ECL Plus Western Blotting detection reagents (GE Healthcare, Buckinghamshire, UK). The image of the signal was exposed to High performance chemiluminescence film (GE Healthcare).

### Cell viability and proliferation assay

#### Cell viability (crystal violet)

Cells were plated onto 6-well plate at 150 000 cells per well in triplicate. After cells had adhered they were serum starved 24 h before they were exposed to the blocker BRL 44408 10^−5^ M (code: 1133; Tocris, Bristol, UK) for 30 min which was followed by exposure to clonidine 10^−6^ M (code C7897; Sigma, Saint Louis, MO, USA) for 24 h.

Cell culture media were removed, and the cells were rinsed before fixed in 1% glutaraldehyde for 30 min and thereafter stained with 0.1% crystal violet (code: C3886; Sigma, Saint Louis, MO, USA) for 30 min. After careful rinsing and air-drying the cells, 30% methanol and 10% acetic acid were added to dissolve the dye. The absorbance was read at 590 nm.

#### Cell proliferation [5-Bromo-2′-deoxy-uridine (BrdU) cell proliferation assay]

The BrdU assay (code: 11299964001; Roche) incorporates BrdU into newly synthesized DNA strands of actively proliferating cells. For the experiment, serum-starved cells were preincubated with the blocker, BRL 44408 10^−5 ^M for 30 min prior to the exposure to clonidine. Analysis followed after 4 h of clonidine 10^−6 ^M incubation.

The protocol was followed according to the manufacturer's instructions. Briefly, cells were incubated with BrdU at 37 °C in a humidified atmosphere of 5% CO_2_ in air. At the end of the incubation cells were washed and fixed. Anti-BrdU antibody was then added and followed by incubation with anti-mouse-Ig-alkaline phosphate. Finally, cells were covered with the substrate solution before mounted in vectashield hard set medium with DAPI (Vector Laboratories H-1500). Results are presented as a ratio of proliferative cells divided with all viable cells from triplicates of each groups.

### Statistics

All statistical calculations were done by the use of computer software (PASW Statistics 18.0.0; SPSS Inc., Chicago, IL, USA). Significance was predetermined at *P* < 0.05.

#### *In vivo* model

To test for significant differences between the different parts of the same tendon, the Friedman test was applied. When comparing the two tendons from the same rabbit, the Wilcoxon Signed Rank test was performed. To compare the mean number of tendon cells in-between the different groups, the Kruskal-Wallis test was performed followed by pair-wise comparisons by way of the Mann-Whitney *U*-test; *P*-values being corrected for the number of tests performed, and these corrected values are presented in the “Results” section.

#### *In vitro* model

Statistical tests used [one-way analysis of variance (ANOVA), followed by Bonferroni post hoc test] are accounted for in the “Results” section, when the test in question is applied. All results were successfully repeated at least once.

## Results

### *In vivo* model

#### General morphology

In the tendon tissue proper from the animals of the clonidine-and NaCl-injected groups tendinosis-like features, similar to those previously described for animals that had exercised for 3 weeks or more in the protocol (Andersson et al., 2011b), was noticed, i.e., abnormally shaped tenocytes, irregular collagen formation, and separation of collagen bundles. This was only rarely noted for the uninjected controls and the BRL (antagonist) injected animals.

#### TH and α_2A_ AR

**Fig 1:**
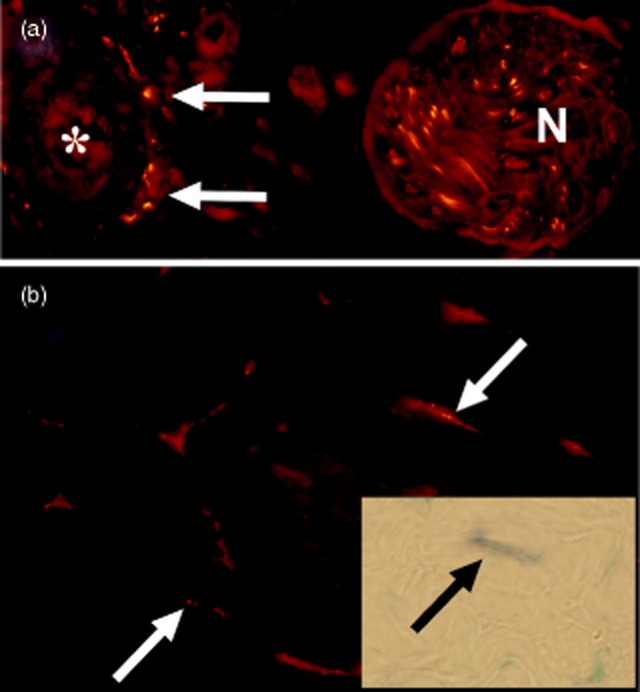
Section of rabbit Achilles tendon tissue processed with immunohistochemistry (a, b; tetramethylrhodamine isothiocyanate staining) and *in situ* hybridization (inset in b; anti-digoxigenin alkaline phosphatase immunostaining detection), for detection of tyrosine hydroxylase (TH; a), alpha-2A-adrenoreceptor (α_2A_ AR; b) and TH mRNA (inset in b). (a) TH-positive nerve structures are seen in the paratendinous tissue in the form of perivascular nerves (arrows) and in a nerve fascicle (N). Asterisk marks lumen of vessel. (b) Tenocytes of the tendon tissue proper display immunoreactions for α_2A_ AR (arrows). (Inset) A tenocyte is positive for TH mRNA (arrow). Antisense staining.

The rabbit Achilles tendon tissue was found to contain nerves immunopositive for TH, especially in the paratendinous tissue. The nerves were seen as perivascular innervation or in nerve fascicles (Fig. 1a). Tenocytes of the tendon tissue proper were found to contain TH and TH mRNA (Fig. 1b, inset) as well as to display immunoreactions for the α_2*A*_ AR (Fig. 1b).

The specific immunoreactions were not seen after omission of primary antibodies. The RNA findings of the antisense staining were not reproduced in sense staining.

#### Effects of exogenously administered adrenergic agonist (clonidine)

**Fig 2:**
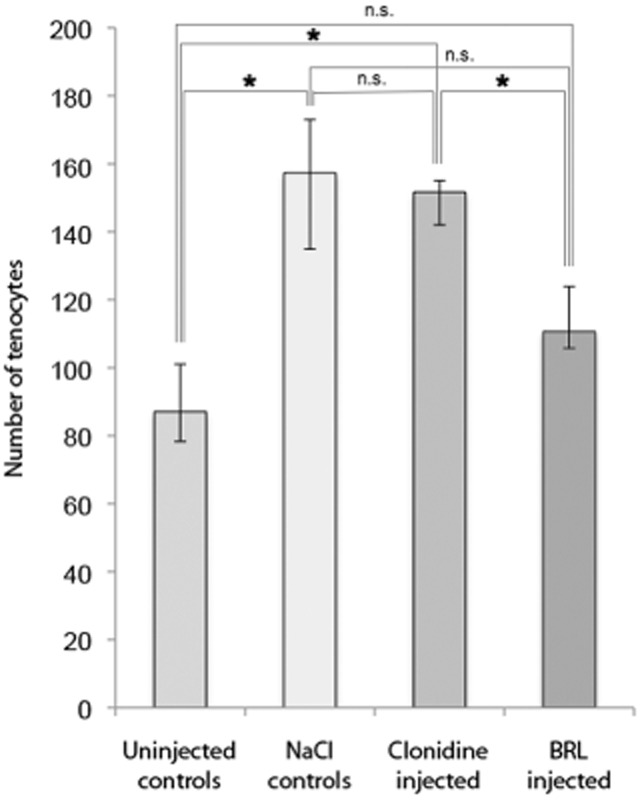
Tenocyte count of the right (exercised) leg of the four groups: Uninjected controls, NaCl-injected controls, animals injected with the α_2_ agonist clonidine, and animals injected with a specific antagonist for the α_2A_ AR (BRL 44408). The median number of tenocytes in the counted area (size 283 × 213 μm; mean of the counted number in three micrographs per tendon part) was significantly (*P < 0.05) increased in the clonidine injected group as compared with the uninjected controls and the antagonist (BRL) injected tendons. The same was seen for the NaCl-injected controls compared with the uninjected controls. No significant difference (n.s.) was found in the comparisons between the rest of the groups. Error bars indicate interquartile range (IQR). Pair-wise Mann-Whitney *U* test was used; *P*-values corrected for the number of tests performed.

There was a significant increase in the number of tenocytes of the tendon tissue proper of the Achilles tendon on the exercised side in the clonidine injected group compared with both the uninjected controls and the animals injected with the adrenergic antagonist (Mann-Whitney *U* pair-wise test; *P* < 0.05, Fig. 2). The NaCl-injected group also showed increased tenocyte number compared with uninjected controls and animals injected with the adrenergic antagonist, although this increase was only significant for the NaCl-injected group vs uninjected controls (*P* < 0.05; Fig. 2).

The median number of tenocytes in the areas of biopsy which were quantified (283 × 213 μm^2^; cf. “Methods”) was 87 for the uninjected controls [with the middle 50% of the observations, i.e., the inter-quartile range (IQR), lying between 78 (Q1 = lowest quartile) and 101 (Q3 = highest quartile)], and 157 for the NaCl controls (IQR: 135–173), see Andersson et al., 2011a, b2011b. The numbers were 152 for the clonidine injected group (IQR: 142–155) and 111 for the BRL injected group (IQR: 106–124).

**Fig 3:**
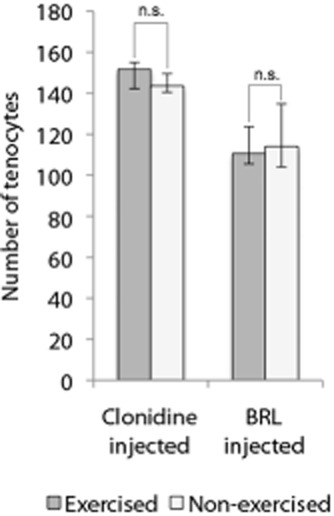
Tenocyte count of the right and left legs of the animals in the clonidine- (α_2_ agonist) and BRL 44408 (α_2A_ AR antagonist) injected groups. No significant difference (n.s.) in the median number of tenocytes could be seen between the exercised (right) and non-exercised (left) legs of the same rabbits. Wilcoxon Signed Ranks test. Error bars indicate interquartile range.

In the clonidine and BRL injected groups, comparisons were made between the tendons of both legs, both of which had been subjected to the injections. No significant differences in the number of tenocytes were seen when comparing the exercised (right) with the non-exercised (left) legs in either group (Wilcoxon Signed Ranks test; *P* #x003E; 0.05, Fig. 3). The non-exercised legs of the animals in the clonidine injected group had a median number of tenocytes per quantified area of 144 (IQR: 141–150) and in the BRL injected group 114 (IQR: 104–135).

No significant differences in vascularity were seen among any of the groups.

### *In vitro* model

#### TH and α_2A_ AR

**Fig 4:**
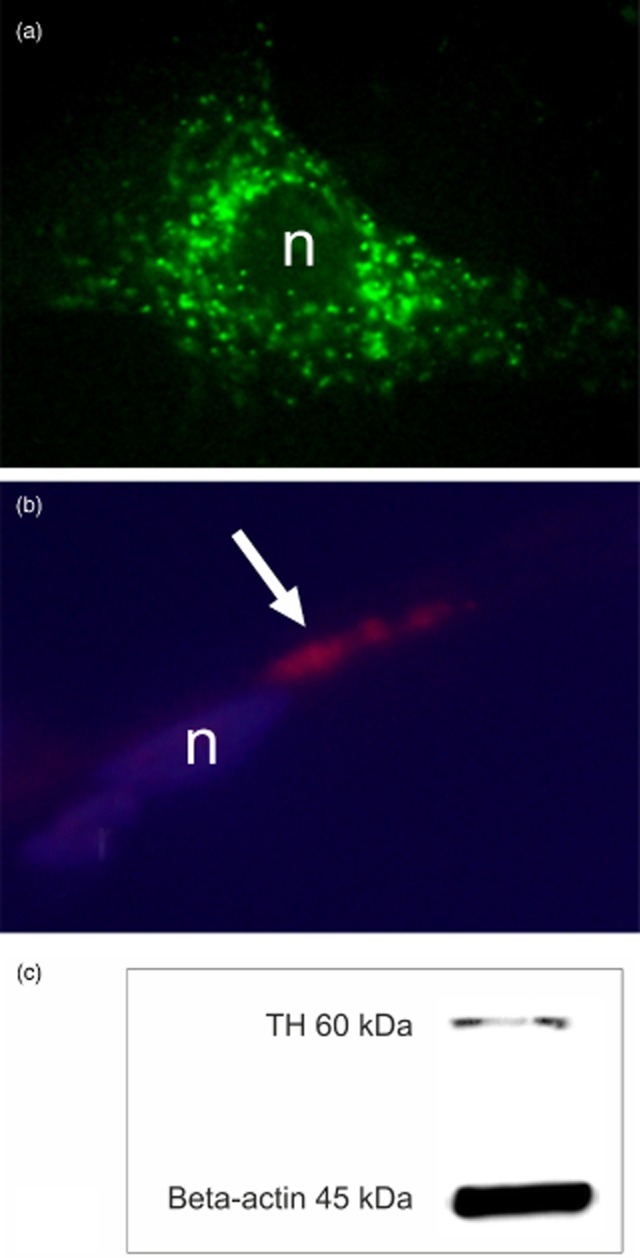
(a, b) Human tendon cells from primary cultures processed with immunocytochemistry. The cell in a is shown to be clearly positive for tyrosine hydroxylase (TH; fluorescein isothiocyanate conjugated staining) and the cell in b express the α_2A_ AR (arrows; tetramethylrhodamine isothiocyanate staining). Nucleus is marked with n (4′,6-diamidino-2-phenylindole-stained in b). (c) Result of Western blot on cultured and lysed human primary Achilles tendon cells; gel stained for TH. A band at the expected molecular weight of TH, i.e., 60 kDa, is immunostained. Beta(β)-actin is shown as a reference.

The human tendon cells in primary culture were found to be clearly immunopositive for TH (Fig. 4a) as well as α_2A_ AR (Fig. 4b). Western blot confirmed the presence of TH in the cultured tendon cells (Fig. 4c). An immunopositive band was present at 60 kDa as expected.

#### Effects of exogenously administered adrenergic agonist (clonidine)

**Fig 5:**
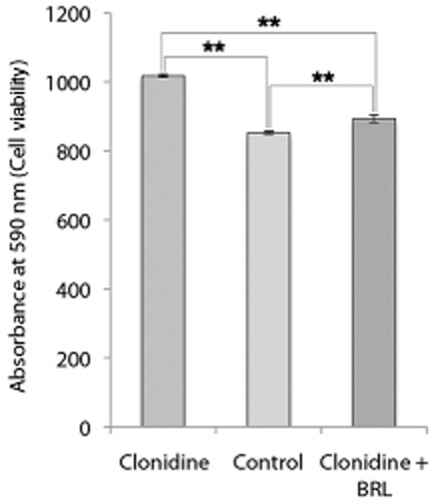
Analysis of viable human tendon cells in primary cultures after 24 h of incubation with the α_2_ agonist clonidine (10^−6 ^M), without clonidine (control), and with clonidine (10^−6 ^M) and the specific antagonist for the α_2A_ AR, BRL 44408 (10^−5 ^M), as measured with crystal violet staining. The significant increase in viable cells seen after incubation with clonidine is effectively reduced with the antagonist. **P < 0.01; error bars indicate standard deviation. (One-way ANOVA; Bonferroni post hoc test).

Clonidine significantly increased the number of viable tendon cells (by close to 20%) after 24 h of incubation as seen by crystal violet staining (*P* < 0.01; One-way ANOVA; Bonferroni post hoc test), and this effect was blocked by simultaneous incubation with the antagonist (Fig. 5).

**Fig 6:**
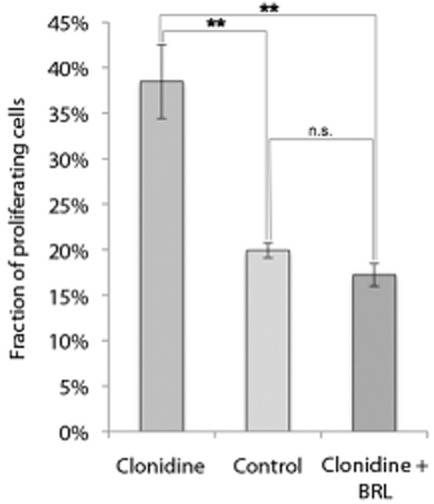
The mean fraction of proliferating (BrdU-positive) human tendon cells in primary cultures after 4 h of incubation with the α_2_ agonist clonidine (10^−6 ^M), without clonidine (control), and with clonidine (10^−6 ^M) and the specific antagonist for the α_2A_ AR, BRL 44408 (10^−5 ^M). The significant increase in the percentage of proliferating cells seen after incubation with clonidine is effectively abolished with the antagonist. **P < 0.01; error bars represent standard deviation. (One-way ANOVA; Bonferroni post hoc test).

The percentage of proliferating (BrdU-positive) tendon cells in cultures after incubation with clonidine was significantly increased (by over 90%) after 4 h (*P* < 0.01; One-way ANOVA; Bonferroni post hoc test), confirming an early activation of proliferation (Fig. 6). Also this effect of clonidine was blocked by the antagonist (Fig. 6).

**Fig 7:**
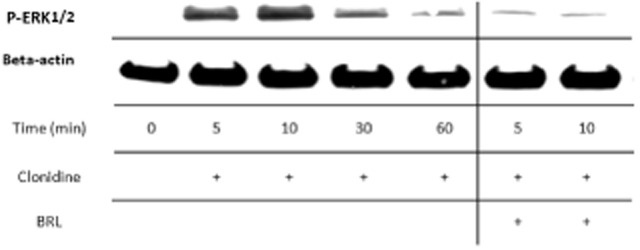
Western blot showing phosphorylated extracellular-signal-regulated kinases 1 and 2 (P-ERK1/2) in cultured human Achilles tendon cells at different time points after incubation with the α_2_ agonist clonidine. The results clearly show that clonidine (10^−6 ^M) activates the phosphorylation of ERK1/2 over time; peeking after 10 min of exposure. The activation by clonidine is effectively blocked when incubated simultaneously with the specific antagonist for the α_2A_ AR, BRL 44408 (10^−5 ^M; result at 5 and 10 min to the right). Beta(β)-actin is shown as a reference.

The Western blot analysis showed that clonidine stimulated phosphorylation of ERK1/2 in the cultured cells; activation peeking after 10 min of exposure (Fig. 7). The ERK1/2 activation by clonidine was effectively blocked when incubated simultaneously with BRL (Fig. 7).

## Discussion

Tenocytes of human tendon tissues express α_2A_ ARs *in vivo*, and this expression is higher in tendinosis patients (Danielson et al., 2007b). Furthermore, human tenocytes themselves seem capable of catecholamine production, a phenomenon unexplained to date, which is also increased in tendinosis patients (Danielson et al., 2007b; Bjur et al., 2008). These findings occur in parallel with a marked paratendinous sympathetic innervation of the larger tendons (Andersson et al., 2007; Danielson et al., 2008). The above observations have led to the hypothesis that adrenergic stimulation might contribute to the tenocyte proliferation that is a prominent tissue feature of tendinosis, and that this effect may be at least partly mediated via activation of α_2A_ ARs on the tenocytes. The present study aimed at testing this hypothesis in an *in vivo* model of Achilles tendon overuse (rabbit) and an *in vitro* human Achilles tendon cell culture model. The results clearly show that stimulation of α_2A_ ARs on tenocytes in both models triggers cell proliferation, thus confirming the hypothesis.

### Adrenergic stimulation increases tenocyte proliferation in rabbit tendon tissue in response to overuse (*in vivo* model)

The study shows that rabbit tenocytes, like their human counterparts, express α_2A_ AR *in vivo*, making them susceptible to adrenergic stimulation, and paratendinous injections of an α_2_ agonist indeed accelerated the development of hypercellularity in the Achilles tendon of the rabbit in response to the overuse protocol during a time period of 1 week. Such changes have previously been shown not to develop until after a minimum of 3 weeks of exercise in this model, if no injections are given (Andersson et al., 2011b). However, as we have earlier demonstrated (Andersson et al., 2011a), a significant increase in cell proliferation was also noted after paratendinous injections of isotonic saline, which is somewhat surprising since isotonic saline would not change the biological micromilieu. The effects of increased local tissue pressure due to 1 mL injections, regardless of injected substance, and the consequent adaptive response (like cellular proliferation) of the tissue must be considered in better understanding these outcomes. Even if potential mechanisms of increased tissue pressure and its effect on the tendon tissue are not yet clarified, the importance of the mere volume increase in injection treatments for tendinosis has previously been discussed (Zeisig et al., 2008). In fact, it has been shown that high volume injections actually reduce tendinosis features like tendon thickness and neovascularization (Humphrey et al., 2010), in part contradicting what is here theorized about adaptive response to increased pressure. Nevertheless, most interestingly, in the present study, injections of 1 mL of a specific α_2A_ AR antagonist clearly inhibited the accelerated tenocyte proliferation, giving evidence for specific α_2A_ AR mediated effects on the observed proliferative response to 1 mL injections. Noteworthy, the injections were given in the area ventral of the Achilles tendon, to which different treatment regimes are directed (Alfredson et al., 2007; Alfredson, 2011) and where sympathetic nerves have been found in great numbers in humans (Andersson et al., 2007).

Of further note, the α_2_ agonist injections, being given in both legs of the rabbit, showed the same proliferative effect on tendon tissue in the unexercised, contralateral leg, as in the tendon of the exercised leg (no significant differences). We have previously demonstrated that the unexercised leg is not a suitable control in this model, as there are bilateral tendinosis changes in response to unilateral training, possibly due to central neuronal mechanisms (Andersson et al., 2011b). However, without injections these changes are only seen after a minimum of 3 weeks of exercise. Thus, the present study demonstrates that adrenergic stimulation leads to tendinosis changes in the rabbit independently from the direct effects of mechanical loading. This leads to the conclusion that adrenergic stimulation might stimulate tenocyte proliferation through pathways distinct from previously identified load-induced responses. Interestingly, from the clinical perspective, it is well known that nonactive individuals, not overloading their Achilles tendons, also suffer from painful midportion Achilles tendinopathy (Alfredson & Lorentzon, 2000).

Concerning the vascularity, no significant differences could be seen between the groups in this study, a finding in sharp contrast to an earlier study on this model testing the effect of the neuropeptide substance P, a substance also found to be produced by human tenocytes (Andersson et al., 2008; Backman et al., 2011). In that study, paratendinous injections of substance P significantly increased intratendinous vascular proliferation (Andersson et al., 2011a), which was not unexpected since substance P has known angiogenic effects (Fan et al., 1993). In contrast, α_2_ adrenergic stimulation by clonidine has been shown to inhibit vascular endothelial growth factor expression in human retinal epithelial cells (Watanabe et al., 2009). In the present animal model, increased vascularity in response to overuse was not noticed until after a minimum of 3 weeks of exercise (Andersson et al., 2011b), and therefore any inhibition of angiogenesis by adrenergic stimulation cannot be evaluated by the design of the present study, which only evaluated the effect of clonidine injections during the course of 1 week of exercise.

Finally, our results suggest that rabbit tenocytes, like human tenocytes, are capable of catecholamine production as evident by the cells expressing TH, giving further evidence of a possible autocrine loop of proliferative regulation, although rabbit tendon tissue, as in humans, was here shown to contain sympathetic nerves as well that might interact with tenocytes via release of norepinephrine. In addition, circulating catecholamines might also act on the adrenoreceptors of the tenocytes.

### Human tenocytes proliferate in response to adrenergic stimulation via activation of MAPKs (*in vitro* model)

The results of the present study suggest that human tendon cells *in vitro* retain the capacity for endogenous catecholamine production (expression of TH) and that they also continue to express the α_2A_ AR. We have previously shown that the vast majority of the cells in this model are of the tenocyte phenotype, retained in the early passages used for our experiments (Backman et al., 2011). The present study furthermore shows that α_2_ stimulation by an exogenously administered specific agonist (clonidine) increases the number of viable tenocytes in culture via augmented cell proliferation, and that this seems to be mediated via phosphorylation of ERK1/2 and initiated already after 5 min of exposure, peaking after 10 min. Preincubation with a specific α_2A_ AR antagonist blocked this response, confirming the specificity of this α_2A_ AR mediated pathway. It is well known that GPCRs like the α_2A_ AR are involved in mitogenic signalling and that activation of MAPKs is one of the involved pathways (New & Wong, 2007). In fact, epinephrine has been shown to increase DNA synthesis via ERK1/2 in mouse embryonic stem cells (Kim et al., 2008), and studies have repeatedly shown that adrenergic α_2_ stimulation regulates proliferation of various cell types, including epithelium (Vazquez et al., 2006), preadipocytes (Bouloumie et al., 1994), and fibroblasts (Seuwen et al., 1990). More specifically, it has been shown that the proliferative α_2_-mediated effects are induced by a rapid increase in the phosphorylation of ERK1/2 (Schaak et al., 2000), which is in accordance with the results of the present study on human tenocytes. It is also interesting to note, that another pathological condition that like tendinosis also involves excessive proliferation of fibroblasts, namely liver fibrosis/cirrhosis, has been shown to be promoted by catecholamines as well, both via the sympathetic nervous system and in an autocrine/self-regulatory fashion through endogenous production of sympathetic transmitters from hepatic stellate cells (Oben & Diehl, 2004).

It should be emphasized that the present study is performed with α_2_ agonist/antagonist which may lead to incomplete activation and inhibition of the relevant catecholamine pathways. Since the present study, as well as earlier tendon studies, has only examined the rate-limiting enzyme in the catecholamine synthesis, TH, any one of the catecholamines might be the most important in tendon pathogenesis (L-DOPA, dopamine, norepinephrine, epinephrine). Nevertheless, the results of this study clearly show that activation of the α_2A_ AR plays a role in the regulation of tenocyte proliferation, making norepinephrine or epinephrine the most plausible candidates. In addition, it should be recalled that other adrenoreceptors than the α_2A_ AR have been found on both human (Danielson et al., 2007b; Bjur et al., 2008) and avian (Wall et al., 2004) tenocytes, such as the α_1_ AR. The functional role of that receptor was not investigated in the present study.

In clinical studies, evaluating the treatment effects after interference with the sympathetic nerve rich paratendinous tissue, it has been shown that tendons that have become pain-free after treatment gradually remodel, and significantly thinner tendons with a more normal structure have been demonstrated in follow-ups (Lind et al., 2006). This might, theoretically, be mediated via a treatment induced blocked adrenergic input, thereby reversing the adrenergic proliferative effects. Also, it has been noticed that ultrasound and Doppler-guided treatments with sclerosing injections (Alfredson et al., 2007) and local scraping (Alfredson, 2011), targeting the regions with vessels and nerves in the paratendinous tissue, are associated with an increased intratendinous blood flow after treatment. This posttreatment local effect on the tendon blood flow could possibly also be seen as an indicator for ongoing local adrenergic influences.

Finally, it is important to conclude, that in the present study, similar results were achieved both in the animal model and in the human cell cultures, which suggests that comparable results can be expected in this kind of research, and that animal models are not necessarily required for all aspects of this research, of importance from and ethical perspective.

## Perspectives

This study clearly shows that α_2_ adrenergic stimulation has a proliferative effect on tendon cells both *in vivo* and *in vitro*. By administration of an α_2A_ AR antagonist, proliferation was inhibited in both models. We propose that this effect can occur during tendinosis pathogenesis, since local catecholamine production in the tendon tissue appears to be upregulated in these patients (Danielson et al., 2007b; Bjur et al., 2008) and since excessive tenocyte proliferation is part of the pathology (Khan et al., 1999). Such effects might also, to some extent, be exerted via peripheral nerves from the autonomous sympathetic nerve system, present particularly around the tendons in question (Andersson et al., 2007; Danielson et al., 2007b, 2008). Interference with the sympathetic nerve rich paratendinous tissue, using sclerosing injections or local scraping, has shown that the tendon gradually remodel and become thinner in patients that has become pain-free after treatment. Treatment with eccentric training also follows the same trend. In theory, the treatments described could possibly mediate their effects via interference with the adrenergic input and thereby reversing the adrenergic proliferative effect, thus leading to tendon healing. Whether interventions directed toward the adrenergic pathway are of prime importance in tendinosis treatment remains to be answered.
